# Physical and Chemical Traits of Grape Varieties Influence *Drosophila suzukii* Preferences and Performance

**DOI:** 10.3389/fpls.2021.664636

**Published:** 2021-04-21

**Authors:** Lisa Weißinger, Katja Arand, Evi Bieler, Hanns-Heinz Kassemeyer, Michael Breuer, Caroline Müller

**Affiliations:** ^1^Department of Chemical Ecology, Bielefeld University, Bielefeld, Germany; ^2^Nano Imaging Lab, Swiss Nano Science Institute, University of Basel, Basel, Switzerland; ^3^Julius-von-Sachs-Institute for Biosciences, University of Würzburg, Würzburg, Germany; ^4^Bavarian State Institute for Viticulture and Horticulture, Veitshöchheim, Germany; ^5^State Institute of Viticulture and Enology (WBI), Freiburg im Breisgau, Germany

**Keywords:** host plants, fruit cuticle, insect behavior, invasive species, physico-chemical properties, spotted wing drosophila, *Vitis* variety, wax composition

## Abstract

The cuticle-covered surface forms the interface between plant parts, including fruits, and their environment. The physical and chemical properties of fruit surfaces profoundly influence plant-frugivore interactions by shaping the susceptibility and suitability of the host for the attacker. Grapevine (*Vitis vinifera*, Vitaceae) serves as one of the various host plants of the spotted wing drosophila, *Drosophila suzukii* Matsumura (Diptera: Drosophilidae), which is invasive in several parts of the world and can cause major crop losses. The susceptibility of wine towards this pest species differs widely among varieties. The objective of our study was to identify physical and chemical traits of the berry surface that may explain the differences in susceptibility of five grape varieties to *D. suzukii*. Both preferences of adult *D. suzukii* and offspring performance on intact *versus* dewaxed (epicuticular wax layer mechanically removed) grape berries were investigated in dual-choice assays. Moreover, the morphology and chemical composition of cuticular waxes and cutin of the different varieties were analyzed. Bioassays revealed that the epicuticular wax layer of most tested grape varieties influenced the preference behavior of adult flies; even less susceptible varieties became more susceptible after removal of these waxes. In contrast, neither offspring performance nor berry skin firmness were affected by the epicuticular wax layer. The wax morphology and the composition of both epi- and intracuticular waxes differed pronouncedly, especially between more and less susceptible varieties, while cutin was dominated by ω-OH-9/10-epoxy-C18 acid and the amount was comparable among varieties within sampling time. Our results highlight the underestimated role of the epicuticular surface and cuticle integrity in grape susceptibility to *D. suzukii.*

## Introduction

Plant-insect interactions have evolved over hundreds of millions of years and are of crucial importance for the earth’s ecosystems (Schoonhoven et al., 2005; Whitney and Federle, 2013). These complex interactions can be mutualistic, commensalistic or antagonistic (Schoonhoven et al., 2005; Calatayud et al., 2018). The latter is of particular relevance for agricultural crop production, because insect pests can cause massive degradation and crop loss. Susceptibility of crop plants to attacks of various herbivorous insect species is known to differ on an inter- but also on an intraspecific plant level (Balagawi et al., 2005; Rebora et al., 2020; Tonina et al., 2020). Most studies focus on differences in leaf traits that influence interactions between plants and herbivorous insects (Robin et al., 2017; Mitra et al., 2020; Saska et al., 2020), while less information is available on physico-chemical properties that drive plant-frugivore interactions (but see, e.g., Rid et al., 2018; Salerno et al., 2020). Plants face a special trade-off in the formation of their fruit properties, as fruits should be attractive for mutualistic dispersers but at the same time unattractive for antagonists such as frugivores and pathogens (Cazetta et al., 2008; Salerno et al., 2020). Variety-specific differences in fruit susceptibility to antagonists are of particular importance in viticulture, where distinct physical and chemical properties determine the sensory characteristics and quality of the wine (*Vitis vinifera*, Vitaceae) (Manso-Martínez et al., 2020; Tonina et al., 2020).

Host plant selection by herbivorous insects is highly complex and influenced by a multitude of physical and chemical traits of their hosts (Schoonhoven et al., 2005). Once insects located their host with the help of visual or olfactory cues (Schoonhoven et al., 2005), characteristics such as fruit skin microstructure and firmness, texture, pulp and fruit consistency, elasticity as well as the chemical composition of the fruit surface influence then host choice and acceptance of frugivores (Rodriguez-Saona et al., 2019; Tonina et al., 2020). Moreover, wax crystals can influence the surface tension and thus impact the attachment of insect tarsae and eggs (Eigenbrode and Espelie, 1995; Müller, 2008; Gorb and Gorb, 2017). Several studies show differences in insect acceptance of fruits of different species and cultivars or varieties and link these to chemical and/or morphological characteristics of epicuticular waxes (Al Bitar et al., 2014; Rid et al., 2018; Rebora et al., 2020) or other specialized metabolites present in the wax layer (Müller, 2006; Shroff et al., 2015). Thus, this first contact zone between the insect and its host is obviously of utmost importance.

The plant surface, the cuticle, is an extracellular membrane with a three-dimensional cutin network originating from the polymerization of C16 and C18 hydroxy fatty acid derivatives (Holloway, 1982). It is associated with a plethora of different wax components comprising predominantly very-long-chain fatty acids and their derivatives as well as cyclic triterpenoids. Waxes are heterogenously distributed across the cuticle with intracuticlar waxes being embedded in the cutin matrix and epicuticular waxes accumulating on the surface (Koch et al., 2004; Müller and Riederer, 2005; Koch and Ensikat, 2008). Within species, the wax composition can differ among organs, across ontogeny and also among varieties (Jetter et al., 2000). Differences in cuticular wax composition among varieties have been found in various crop plants (Leide et al., 2007; Rebora et al., 2020), including *V. vinifera* (Radler, 1965; Pensec et al., 2014). In this species, a high heterogeneity in the fruit cuticle wax was shown, where epicuticular wax crystals solely consist of very-long-chain aliphatic compounds, while the intracuticular wax is dominated by triterpenoids (Arand et al., 2021).

One of the major pests attacking fruits of various crop plants, including vine, is the fruit fly *Drosophila suzukii* Matsumura (Diptera: Drosophilidae; spotted wing drosophila). This frugivorous pest was accidentally introduced to Europe and North America and has become a well-established economic pest in several countries in the recent years (Cini et al., 2012; Asplen et al., 2015; Poyet et al., 2015). The fly’s ability to infest intact, ripening, soft-skinned fruits including grapes, combined with its high fecundity and short development cycle, makes it a severe economic threat, especially for the small-fruit industry (Lee et al., 2011; Weißinger et al., 2019a; Little et al., 2020a). Additionally, lesions in the cuticle caused by oviposition serve as preferred entry sites for microorganisms, leading to secondary infections, e.g., with acetic acid bacteria (Ioriatti et al., 2018; Ebbenga et al., 2021). Since *D. suzukii* females are able to actively pierce the intact skin of ripening fruits for oviposition (Atallah et al., 2014), berry firmness is discussed to play a relevant role in their host selection (Burrack et al., 2013; Rodriguez-Saona et al., 2019). In field and laboratory studies, we previously demonstrated that susceptibility differs within and between certain varieties of *V. vinifera*, with a general preference of *D. suzukii* for red over white varieties and damaged over intact berries (Weißinger et al., 2019a, b). Further investigations are necessary to understand potential cues driving this fruit-frugivore interaction.

In the present study, we assessed several physical and chemical traits of five grape varieties that may influence preferences and offspring performance of *D. suzukii* and characterized the morphology and chemical wax composition of these varieties. We conducted dual-choice bioassays offering berries of different conditions to determine whether removal of the epicuticular wax layers in these varieties may affect the choice behavior and development of *D. suzukii*. Furthermore, we analyzed the berry sugar content, wax morphology, skin firmness as well as the chemical composition of the different cuticular wax components to disentangle factors that may explain the differences in grape susceptibility of the varieties to the flies.

## Materials and Methods

### Insects and Grapes

A breeding stock of the Asian fruit fly *Drosophila suzukii* was established from field-collected adults and maintained for over 50 generations at State Institute of Viticulture and Enology, Germany (as in Weißinger et al., 2019a). The flies were kept in rearing cages (30 cm × 30 cm × 30 cm, Bugdorm-1, Megaview, Taiwan) in a climatic chamber (Viessmann, Allendorf, Germany) under controlled conditions (24°C, 60–70% r.h., L16:D8) and provided with a sugar-water source (5% sucrose) and a food source consisting of a mix of brewer’s yeast (Masterdog, Schwieberdingen, Germany) and dry sugar (1:1). Additionally, Petri dishes (Ø 9 cm) filled with “San Michele medium” (Briem et al., 2016) were placed in the cages as a food source and oviposition medium for 2–3 days. Subsequently, Petri dishes were removed, sealed with perforated lids and kept under the same conditions as the colony until adult emergence. Adults were placed in new rearing cages. The dual-choice assays were conducted with 6–12-day-old adult female flies.

Three red (“Regent”, “Acolon”, and “Pinot Noir”) and two white grape varieties (“Müller-Thurgau” and “Pinot Blanc”) of *Vitis vinifera* were chosen for bioassays and grape characterization (Table 1). Grape berry clusters of these varieties were randomly collected from vineyards in the viticultural district Kaiserstuhl (48° 40 51″ N, 7° 400 14″ E; 556.6 m a.s.l.; Baden-Wuerttemberg, southwestern Germany) in 2016 and 2017. Grapes were harvested at typical variety-specific harvest °Brix (17.9–25.4, Table 1), which corresponds to the BBCH-stage 89 according to Lorenz et al. (1994). Detailed information on selection of investigated varieties, vineyards, berries and storage are described in Weißinger et al. (2019a). Randomly selected berries of the same grape clusters were used to assess behavioral responses of the insects as well as chemical and physical fruit and wax characteristics.

**TABLE 1:** Grape varieties (intact or dewaxed berries) that were offered to *Drosophila suzukii* females for 24 h in dual-choice arena bioassays.

Test	Grape varieties	Replicates (arenas)	Year	Brix
1	Regent	10	2016	18.6°
		10	2016	20.9°
		10	2017	19.4°
2	Acolon	10	2016	17.9°
3	Pinot Noir	10	2016	25.4°
		10	2017	23°
4	Müller-Thurgau	10	2017	18.4°
5	Pinot Blanc	10	2017	20°

*Relative density (in °Brix) of ten pooled and crushed berries of the same grape bunches as used in choice bioassays.*

### Insect Bioassays

To investigate whether varieties may turn more preferred and susceptible when epicuticular wax crystals are damaged or removed, intact berries (“intact”) and berries, from which epicuticular wax layers had been mechanically removed (“dewaxed”), were offered to *D. suzukii* in dual-choice assays. For removal of epicuticular waxes, half of the grape berries of each variety were carefully rubbed with tissue. To test overall acceptance of the grape varieties a small sample of intact berries of each variety was offered to *D. suzukii* in the rearing cages and as soon as infestation took place dual-choice assays were started. Ten female flies per test arena (19 cm diameter glass dish covered with gauze lid) were given a choice among intact and dewaxed berries of the same variety, offered each in two groups of three berries placed in sand in Petri dishes (6 cm diameter; for details see Weißinger et al., 2019a; Supplementary Figure 1). For each grape variety, dual-choice assays were replicated 10–30 times in 2016 and 2017 (Table 1). After 5, 7, and 24 h the position of all individuals in the test arenas was recorded (location preference). In addition, after 24 h the number of successfully deposited eggs, showing vertically inserted and visible egg filaments (Supplementary Video 1), was counted (oviposition preference) under a microscope (Zeiss Discovery V20, Jena, Germany). To test offspring performance, all berries used in the dual-choice assays were placed in separate plastic cups (500 mL) and stored in the climate chamber. Emergence of adults was recorded every 5–7 days for about 30 days for all tests conducted in 2017.

### Berry Parameters

#### Sugar Content

Sugar content (°Brix) of each variety was determined by pressing the juice of ten randomly selected berries on a handheld, temperature-compensated refractometer (VWR, Darmstadt, Germany) within 24 h after collection of the berries in the field (Table 1).

#### Berry Skin Firmness

**FIGURE 1:**
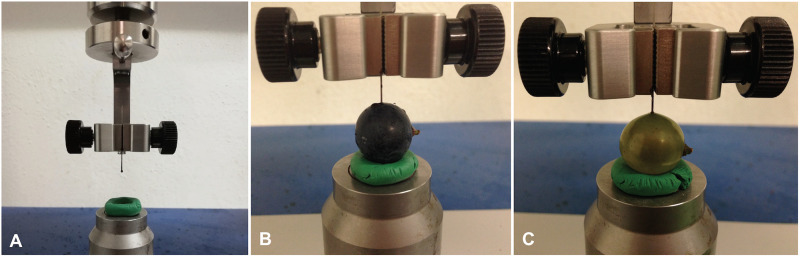
Puncture test measurement of grape berries. **(A)** Universal Testing Machine (Inspekt table blue 5, Hegewald and Peschke Meß- und Prüftechnik GmbH) modified with a ring made from plasticine and an insect pin (No. 3, Elephant Brand, Austria). **(B)** Intact berry of “Regent”. **(C)** Dewaxed berry of “Pinot Blanc”.

Berry skin firmness of intact berries and dewaxed berries was measured from 5–11 intact berries per variety and experimental date within 24 h after harvest in 2017. As “Acolon” was only included in tests in 2016, no firmness data are available for this variety. Berry skin firmness was measured as maximum penetration force (Fmax in cN) using the puncture test technique adapted from Letaief et al. (2008) on a Universal Testing Machine (Inspekt table blue five, Hegewald and Peschke Meß- und Prüftechnik GmbH, Nossen, Germany) (Figure 1). Therefore, single berries were placed with the pedicel in a horizontal orientation on the metal plate of the machine. The machine was equipped with a blunted insect pin (No. 3, 38 mm × 0.50 mm, Elephant Brand, Austria) with the ball of the pin facing downwards to mimic a puncture created by the serrated ovipositor of *D. suzukii*. A ring made from plasticine was used to keep the berries in place (Figure 1). Puncture measurements were conducted once along the equatorial region of each berry and Fmax that a needle probe moving at 1 mm/s needed to puncture the berry skin was taken.

#### Scanning Electron Microscopy of Berry Surface

The wax surface structures were visualized with a scanning electron (SE) microscope (Philips XL30 ESEM) equipped with a cryo-preparation unit (Alto 2500, Gatan, United Kingdom). Intact slices of the berry skin were fixed with low temperature glue on a specimen holder without impairing the waxy surface. Cryo-fixation with a nitrogen slush (≤185°C) was followed by sputtering with 20 nm Au in a high vacuum cryo-preparation chamber. The sputtered and frozen specimen were examined with a SE detector operating with acceleration voltage of 5–10 kV at high vacuum and −150°C. The images were acquired and documented with DISS5 Software from REM-X GmbH Bruchsal.

#### Wax and Cutin Analysis

For chemical analysis of waxes, grape berries were examined under the dissecting microscope to exclude pre-infestation and to discard visibly damaged and overripe berries. Fruits were carefully washed with deionized water and air-dried. A spherical shape was assumed for all fruits and the surface area was calculated from the average of the longitudinal and transverse diameter. Epicuticular wax crystals were obtained by rubbing the entire fruits with KimtechScience precision wipes (Kimberly-Clark, Koblenz, Germany) until they appeared shiny. Two fruits were used for one sample and five samples were analyzed in total. The wipes were extracted three times with chloroform and *n*-tetracosane was added as internal standard.

After removal of epicuticular waxes, disks (7 mm in diameter, ∼2 mm depth) were cut from the polished fruits with a cork borer and incubated in enzyme solution [1% Trenolin Super DF (Erbslöh, Geisenheim, Germany) and 1% celluclast (Novo Nordisk, Mainz, Germany) in 0.1 M citric acid with 0.01 M sodium azide] until the cuticle was fully separated from the tissue. Isolated cuticular membranes were extensively washed with deionized water, air dried and extracted 10 min in chloroform to obtain the remaining intracuticular waxes. The chloroform was evaporated and the residue was dissolved three times in methanol and filtered to obtain the more polar wax compounds (as in Staiger et al., 2019). The residue in the filter was extracted three times with chloroform to dissolve the more lipophilic wax fraction. *n*-Tetracosane was added to the methanol and chloroform phases as internal standard. Three randomly selected discs were used for one sample and five replicates were analyzed.

The dewaxed cuticular membranes were transesterified with boron trifluoride in methanol (Fluka, Seelze, Germany) over night at 70°C. Chloroform, *n*-dotriacontane (C32, Sigma-Aldrich, Darmstadt, Germany) as internal standard and sodium chloride-saturated aqueous solution (AppliChem, Darmstadt, Germany) were added. Methyl-esters of the de-esterified cutin monomers were extracted from this two-phase system three times with chloroform. The combined chloroform phases were dried over anhydrous sodium sulfate (AppliChem, Darmstadt, Germany) and filtered.

All wax and cutin samples were evaporated to dryness under a flow of nitrogen. *N*,*O*-bis(trimethylsilyl)trifluoroacetamide (BSTFA, Marchery-Nagel, Düren, Germany) in pyridine (Merck, Darmstadt, Germany) was used for 30 min at 70°C to convert reactive functional groups into trimethylsilyl derivatives. Gas chromatography coupled with mass spectrometry or flame ionization detection were used for qualitative and quantitative determination of the single wax and cutin compounds, respectively, following the settings as described in Leide et al. (2007). The total amount of single wax compounds in the intracuticular wax was calculated by adding the respective amounts in the methanol and chloroform fraction. All values were normalized to the internal standard and the surface area (μg cm^–2^).

### Data Analysis

The program R, version 3.5.1 (R Development Core Team, 2018), was used for all statistical analyses and figures. To analyze the effects of grape berry condition (intact *versus* dewaxed) on location and oviposition preferences of *D. suzukii*, separate linear mixed effects models (LMMs) with a Gaussian error distribution were performed for each test combination [R package: lme4 (Bates et al., 2014)]. In order to choose the appropriate error distribution (“family”) for LMMs, it was assured that residuals exhibited variance homogeneity and normal distribution of residuals by means of visual inspection (Zuur et al., 2009). We applied step-wise backwards model selection based on chi-square likelihood ratio tests to obtain *P*-values for the effects of all predictor variables (Venables and Ripley, 2000).

For statistical analyses of the location preference data, the number of flies sitting on or within a radius of <1 cm around the berries of the same berry condition (6 berries per arena) after 24 h was summed up for each test arena. We calculated separate LMMs for each grape variety, which comprised the number of *D. suzukii* females per choice option as response, the factor berry condition as fixed effect and the test arena as well as year as random effects. For the oviposition preference data, the number of eggs in berries of the same choice option (berry condition) was calculated per arena. For each grape variety, a separate LMM was performed, which comprised the number of eggs per choice option as response, the factor berry condition as fixed effect and the test arena as well as year as random effects. For statistical analyses of the adult emergence data of 2017, we calculated the proportion of emerged adults from deposited eggs per berry condition and compared these using a Mann–Whitney *U* test.

The Fmax values of intact *versus* dewaxed berries within a variety were analyzed using a Mann–Whitney *U* test. To visualize the chemical composition of the intra- and epicuticular wax compounds of all varieties, a principal component analysis (PCA) was performed. Therefore, zeros were replaced by very low random numbers (between 10^–13^ and 10^–12^) and data autoscaled (mean-centered to unit variance).

## Results

### Influences of Epicuticular Wax Layers on Fly Preferences and Performance

**FIGURE 2:**
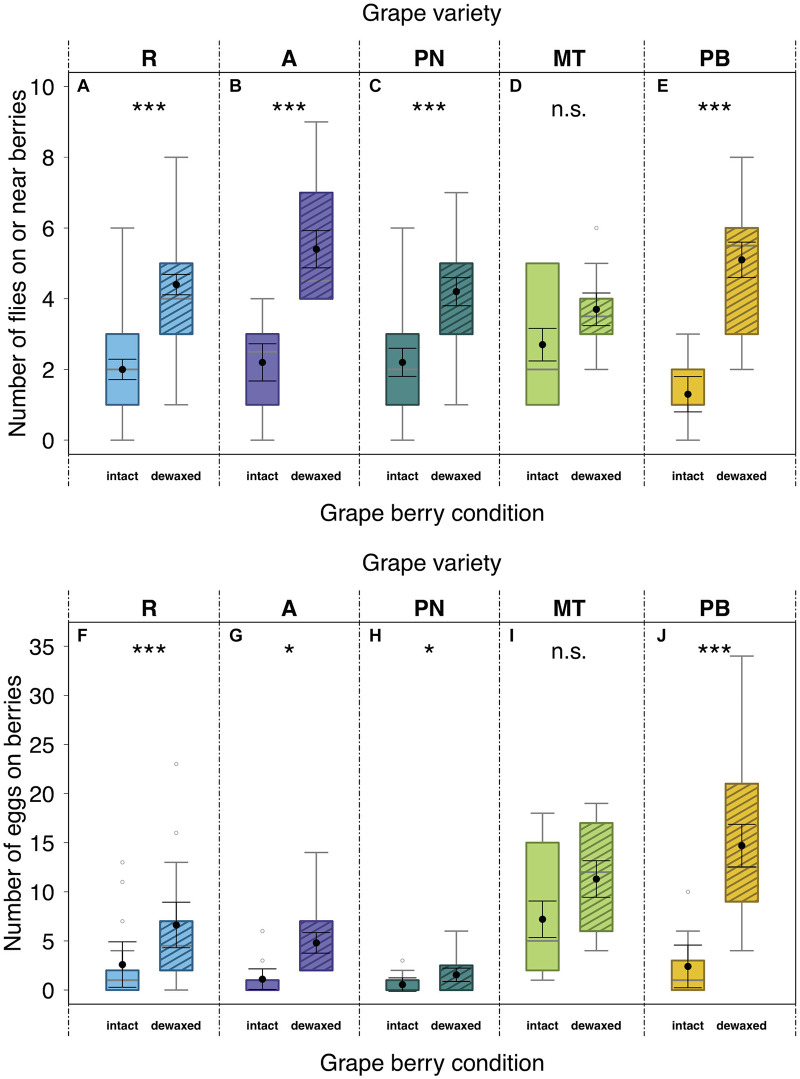
Effects of grape berry condition on preferences of *Drosophila suzukii*. Fly number on or near berries within 1 cm radius (location preference; **A–E**) and the number of laid eggs per grape berry condition (oviposition preference; **F–J**) in dual-choice bioassays after 24 h. Adult female flies (*n* = 10 per arena) were given the choice between berries with intact waxes (intact) or epicuticular waxes removed (dewaxed) of “Regent” (R) (*n* = 30), “Acolon” (A) (*n* = 10), “Pinot Noir” (PN) (*n* = 20), “Müller-Thurgau” (MT) (*n* = 10), and “Pinot Blanc” (PB) (*n* = 10). Asterisks denote significant differences (n.s., not significant; *P* > 0.05), *P < 0.05, ***P < 0.001) between tested berry conditions (analyzed with LMMs; for statistical results see Table 2). Medians (gray lines) are presented with interquartile ranges (boxes), whiskers extend to the maximum and minimum values within the 1.5-fold interquartile range and outliers are shown as open circles. Black dots with error bars represent the least square means with their standard errors extracted from the LMMs, accounting for the specific error distribution of the response factor and for random effects.

In dual-choice bioassays offering intact and dewaxed berries of the same variety, 66% of the test females had made a choice for one of the offered berry conditions within 24 h. Significantly more flies were sitting on or near dewaxed over intact berries (= location preference) of “Regent”, “Acolon”, “Pinot Noir”, and “Pinot Blanc” (Figures 2A–C,E and Table 2), whereas in assays with “Müller-Thurgau” flies showed no significant location preference (Figure 2D and Table 2). Likewise, significantly more eggs were found on dewaxed berries than on intact berries of most varieties except ‘Müller-Thurgau’ (Figures 2F–I and Table 2). The number of deposited eggs was about three (“Regent” and “Pinot Noir”) up to six times (“Pinot Blanc”) higher on the dewaxed compared to the intact berries.

**TABLE 2:** Influences of berry condition of *Vitis vinifera* (intact or dewaxed) on *Drosophila suzukii* behavioral (location and oviposition) preferences in dual-choice assays.

Grape variety		Regent		Acolon		Pinot Noir		Müller-Thurgau		Pinot Blanc	
Fixed effects	*Df*	*X^2^*	*P*	*X^2^*	*P*	*X^2^*	*P*	*X^2^*	*P*	*X^2^*	*P*
**Location preference**											
Berry condition	1	26.97	**<0.001**	14.10	**<0.001**	11.47	**<0.001**	2.46	0.117	19.14	**<0.001**
Random effects		Var	Obs	Var	Obs	Var	Obs	Var	Obs	Var	Obs
ID		0	30	0	10	0	20	0	10	0	10
Year		0	2			0	2				
Residuals		2.4	60	2.5	20	3.17	40	1.91	20	2.5	20
**Oviposition preference**											
Berry condition	1	12.47	**<0.001**	5.93	**0.015**	5.95	**0.015**	2.72	0.099	12.73	**<0.001**
Random effects		Var	Obs	Var	Obs	Var	Obs	Var	Obs	Var	Obs
ID		0	30	0	10	0	20	4.34	10	0	10
Year		4.4	2			0	2				
Residuals		17.5	60	9.93	20	1.6	40	26.85	20	47.25	20

*X^2^- and P-values for the fixed effect term obtained from likelihood ratio tests on linear mixed-effects models (LMMs) (P-values < 0.05 are highlighted in bold) and estimates for the variance (Var) explained by random effects as well as unexplained variance (residuals) with the number of observations (Obs) for each of these groups.*

The emergence rate of fly offspring hatching from dewaxed “Regent” berries was about five times and thus significantly higher from eggs deposited in dewaxed than in intact berries of this variety (Table 3). From the low number of eggs laid on “Pinot Noir” berries, no adults emerged at all. There were no significant differences in adult emergence rate from eggs deposited in berries of intact vs. dewaxed “Müller-Thurgau” or “Pinot Blanc” (Table 3). Overall, emergence rates were quite low, with a maximum of about 48% from dewaxed “Pinot Blanc” berries and a maximum of 27% from intact berries of “Regent”.

**TABLE 3:** Mean (±SD) adult emergence rate *of Drosophila suzukii* from eggs laid in berries of *Vitis vinifera* of different condition (intact or dewaxed) in dual-choice assays performed in 2017 (see Table 1); *P*-values < 0.05 are highlighted in bold (Mann–Whitney *U* test), *N* = 10.

Grape variety	Berry condition	Emergence rate (%)	*W*	*P*
Regent	Intact	4.2 ± 9.3	21	**0.018**
	Dewaxed	17.5 ± 11.8		
Pinot Noir	Intact	0 ± 0	50	1
	Dewaxed	0 ± 0		
Müller-Thurgau	Intact	4.8 ± 6.7	42.5	0.553
	Dewaxed	9.0 ± 12.9		
Pinot Blanc	Intact	0 ± 0	30	0.087
	Dewaxed	10.2 ± 18.4		
				

### Influences of Epicuticular Wax Layers on Berry Firmness

**FIGURE 3:**
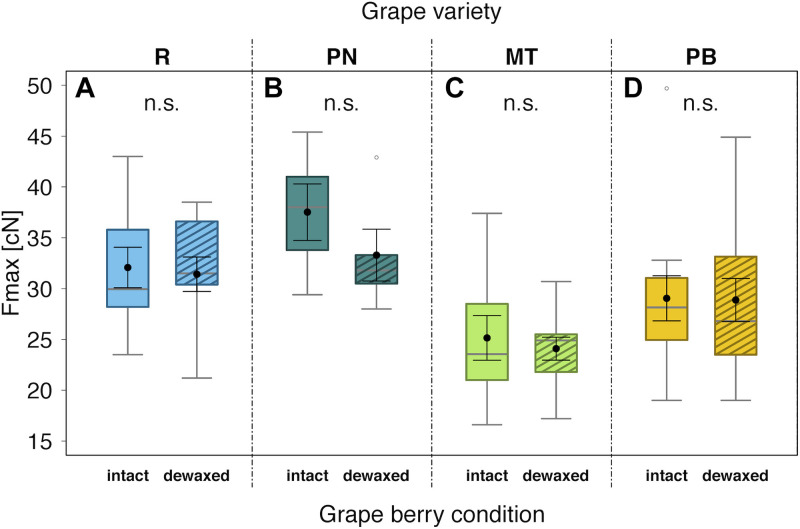
Maximal penetration force (Fmax) to puncture the skin of berries with intact waxes (intact) or epicuticular waxes removed (dewaxed) of *Vitis vinifera* varieties “Regent” (R) **(A)** (*n* = 10), “Pinot Noir” (PN) **(B)** (*n* = 5), “Müller-Thurgau” (MT) **(C)** (*n* = 10), and “Pinot Blanc” (PB) **(D)** (*n* = 12) in 2017; n.s., not significant (*P* > 0.05) (analyzed with Mann–Whitney *U* test). Medians (gray lines) are presented with interquartile ranges (boxes), whiskers extend to the maximum and minimum values within the 1.5-fold interquartile range and outliers are shown as open circles. Black dots with error bars represent the means with their standard errors.

The mean Fmax values, used as measure of berry firmness, ranged between 24.1 cN for “Müller-Thurgau” and 37.5 cN for “Pinot Noir” (Figure 3). The berry firmness did not differ significantly between intact and dewaxed berries for any of the four tested grape varieties.

### Morphology of Epicuticular Waxes of Grape Berries

**FIGURE 4:**
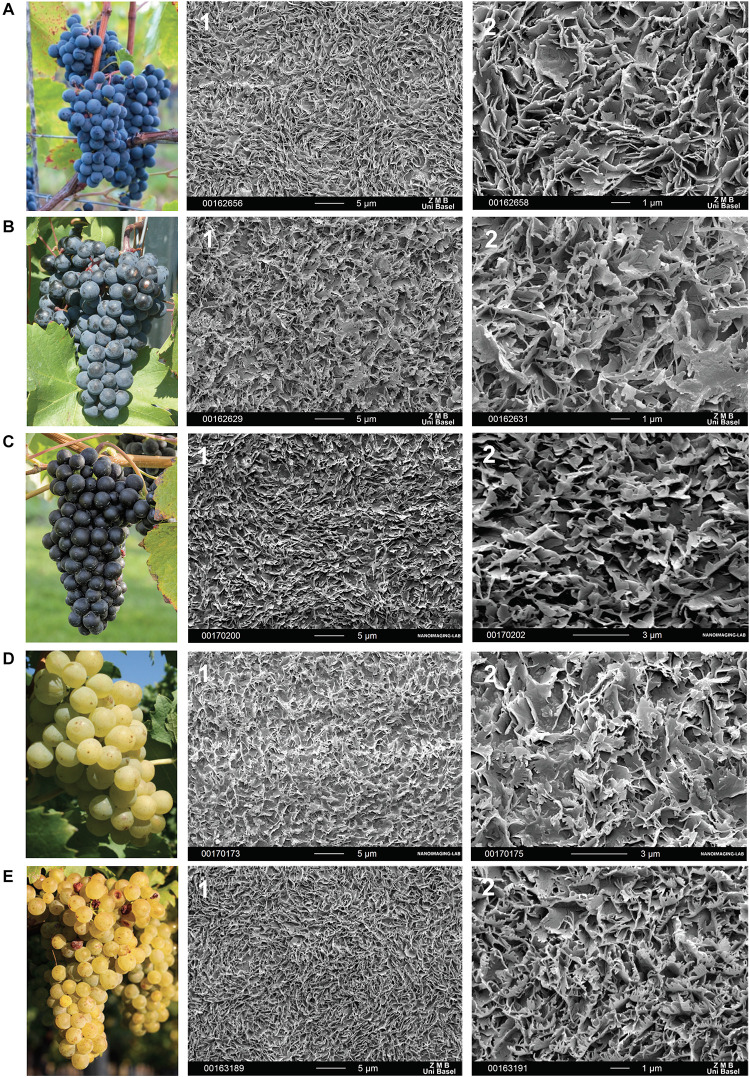
Morphology of *Vitis vinifera* varieties **(A–E)** and respective surface of a berry in full maturity with epicuticular wax crystals recorded with cryo-scanning electron microscopy at two magnifications (1) 2,500X and (2) 8,000X. Red *Vitis vinifera* varieties **(A)** “Regent”, **(B)** “Acolon”, and **(C)** “Pinot Noir” as well as white cultivars **(D)** “Müller-Thurgau” and **(E)** “Pinot Blanc”.

In the cryo scanning-electron microscope, the berry surfaces appeared more or less frosted (Figure 4) which is likely caused by the formation of three-dimensional epicuticular wax crystals. Epicuticular wax crystals on the berry surfaces build a dense layer of irregular platelets with different fine structures (Figure 4). In “Regent” (Figure 4A), sharp-edged, serrated platelets protruded almost vertically in swirl-like formations, while platelets in “Pinot Noir” (Figure 4C) appeared more smooth with rounded edges. In “Acolon” (Figure 4B), “Müller-Thurgau” (Figure 4D) and “Pinot Blanc” (Figure 4E), platelets appeared more filamentous and more or less molten at the base.

### Composition of Cuticular Waxes and Cutin From Grape Berries

**FIGURE 5:**
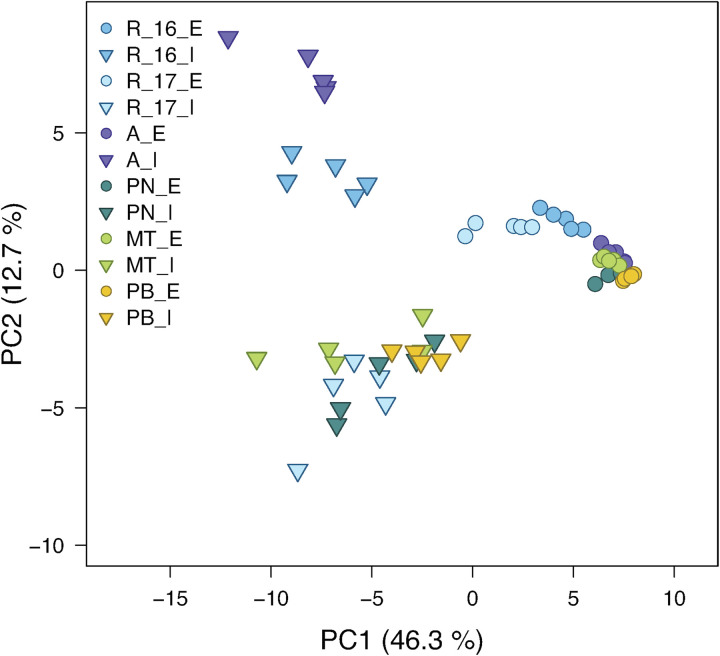
Principal component analysis of composition of epicuticular (E; circles) and intracuticular (I; triangles) waxes (total number of wax compounds = 87) of the *Vitis vinifera* varieties “Regent” (R) [measured in 2016 (R_16) and 2017 (R_17)], “Acolon” (A), “Pinot Noir” (PN), “Müller-Thurgau” (MT) and “Pinot Blanc” (PB) (*n* = 5 per variety). The percent of total variance explained by each PC is given in brackets.

**FIGURE 6:**
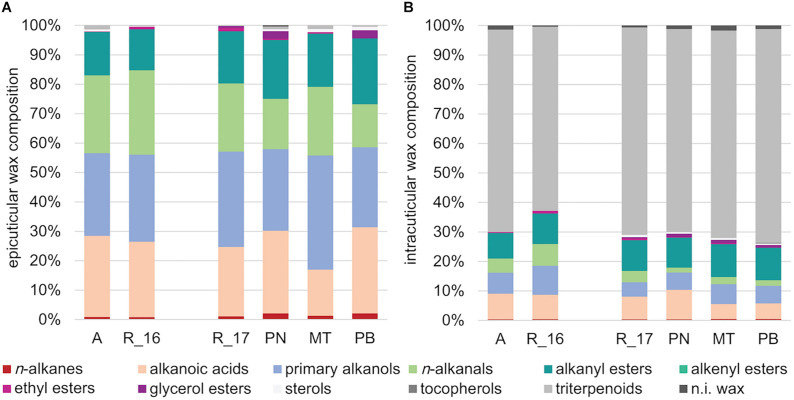
Wax composition of **(A)** epi- and **(B)** intracuticular waxes (in %) of the *Vitis vinifera* varieties “Acolon” (A), “Regent” (R_16 in 2016 and R_17 in 2017), “Pinot Noir” (PN), “Müller-Thurgau” (MT), and “Pinot Blanc” (PB) (*n* = 5 per variety and year).

In a principle component analysis (PCA), the overall composition of both epi- and intracuticular wax compounds was quite distinct between the varieties, with the strongest separation between “Acolon” and “Regent” versus the other three varieties (Figure 5). The wax composition also differed between “Regent” berries collected in 2016 and 2017. The epicuticular wax portion, which could be removed mechanically, amounted to less than 5% of the total wax coverage per surface area, except for “Regent” with 11.4% in 2016 and 18.2% in 2017 (Table 4). Epicuticular waxes were almost entirely composed of very long chain aliphatic acids, alkanols, alkanals and esters, while significant amounts of triterpenoids were missing in all varieties (Figure 6).

**TABLE 4:** Mean (SD) wax amounts and cutin composition of grape berry cuticles from ripe fruits of *Vitis vinifera* varieties “Regent” (R), “Acolon” (A), “Pinot Noir” (PN), “Müller-Thurgau” (MT), and “Pinot Blanc” (PB) (*n* = 5 per variety and year).

	*Vitis vinifera* 2016		*Vitis vinifera* 2017			
	R	A	R	PN	MT	PB
**Epicuticular wax**						
Wax load (μg cm^–1^)	9.7 (2.0)	3.5 (1.3)	16.2 (2.8)	2.9 (0.9)	4.0 (0.8)	2.2 (0.6)
Aliphatic fraction (%)	100	98	100	98	98	99
Cyclic fraction (%)	>1	2	>1	2	2	2
Main compounds	mix of alcohols, fatty acids, aldehydes, alkyl esters					
**Intracuticular wax**						
Wax load (μg cm^–1^)	107.6 (9.6)	113.4 (5.8)	96.1 (8.7)	91.9 (4.8)	105.1 (13.1)	81.1 (6.0)
Aliphatic fraction (%)	30	28	29	28	28	26
Cyclic fraction (%)	70	72	71	72	72	74
Main cyclic compound	oleanolic acid					
Main cyclic compound (% of cyclics compound)	89	79	93	92	88	92
**Cutin**						
Cutin load (μg cm^–1^)	87.3 (9.0)	59.3 (4.3)	50.3 (3.7)	45.0 (2.4)	52.2 (3.6)	48.0 (3.4)
Main compound	ω-OH-9/10-epoxy-C18					
Main compound (%)	39	37	42	41	45	39
C16:C18 ratio	1:4.8	1:9.3	1:3	1:4.5	1: 2.8	1:4.3
Degree of unsaturation (%)	26	28	16	19	14	23
ACL	17.9	18.2	18.3	18.4	18.2	18.7
Total wax:cutin ratio	1.3:1	2:1	2.2:1	2.1:1	2.1:1	1.7:1

*Wax amounts of epicuticular waxes were determined from intact berries. Remaining intracuticular waxes and cutin were analyzed using isolated cuticles of the same berries. ACL, average chain length.*

In contrast, the intracuticular waxes were dominated by cyclic triterpenoids with oleanolic acid representing the main fraction (Figure 6 and Table 4). The remaining 26–30% also consisted of very long chain aliphatics, similar to the epicuticular waxes. The amount of cutin monomers (∼50 to 60 μg cm^–1^) was found to be about half of the amount of total waxes for all varieties, except for “Regent” in 2016 with a much higher cutin load (∼87 μg cm^–1^). All varieties exhibited a mixed C16/C18 cutin type with higher amounts of octadecanoic acid derivatives and ω-hydroxy-9,10-epoxy-octadecanoic acid as main compound (Table 4). The degree of unsaturation was lowest in “Müller-Thurgau” (14%) and highest in “Acolon” (28%), whereas the average chain length was around 18 carbon atoms in all varieties (Table 4).

## Discussion

Dual-choice assays revealed that *D. suzukii* preferences were clearly affected by berry surfaces for almost all tested grape varieties (Figure 2 and Table 2). Overall, more flies rested and deposited more eggs on berries, whose epicuticular wax layers were removed (dewaxed), compared to intact berries except of “Müller-Thurgau” (Figures 2D,I). When berries start to touch each other during development, their wax coat appears patchier and glossy spots become visible (Arand et al., 2021). Thus, parts of epicuticular wax crystals may also be reduced under natural conditions, causing potentially higher acceptance by this fly species. Previous field observations indicated that grape berries of rather less accepted varieties, even if not obviously pre-damaged, can still be attacked sporadically by *D. suzukii* (Weißinger et al., 2019a). Nevertheless, *D. suzukii* females prefer damaged (artificial damage of the surface, causing a 2–3 mm wide wound) over intact grape berries (Weißinger et al., 2019a), as similarly also found for fruits of various other plant species (Stewart et al., 2014; Holle et al., 2017). Thus, changes in physical and chemical traits of fruit surfaces, e.g., due to a missing or disrupted epicuticular wax layer, clearly influence the susceptibility of fruits to *D. suzukii* as further discussed below. Several studies found a negative correlation between skin firmness of single berries and *D. suzukii* oviposition preference, i.e., an increased infestation with decreasing fruit firmness (Lee et al., 2016; Shrader et al., 2018; Entling et al., 2019). In contrast, other studies have shown the opposite (Rodriguez-Saona et al., 2019), or even revealed no clear correlation between the two parameters (Stewart et al., 2014; Andreazza et al., 2016; Little et al., 2017). In line with the latter results, we likewise observed no relationship between oviposition preference and skin firmness in the present study. Within the grape varieties investigated, skin firmness did not differ between intact and dewaxed berries (Figure 3), being thus not necessarily the deterministic factor for *D. suzukii* oviposition preference. This observation supports previous findings of firmness of more and less susceptible grape cultivars (Weißinger et al., 2019a). Skin firmness is only one of the complex anatomical and physical berry characteristics. Other factors, such as the microstructure, texture, thickness, consistency and elasticity of the pulp and berries also influence the susceptibility to *D. suzukii* (Tonina et al., 2020).

The overall emergence rate of offspring was very low in all tested grape varieties (Table 3), in line with previous findings for grape (Weißinger et al., 2019a) and no offspring emerged from berries of “Pinot Noir” at all. In accordance with other studies (Lee et al., 2011; Linder et al., 2014), we can thus reconfirm that grapes do not provide a very suitable environment for larval development. However, in the present study considerably more offspring emerged from eggs deposited in dewaxed than in intact berries at least in the red variety “Regent” (Table 3). In contrast, significantly higher offspring numbers emerging from artificially pre-damaged compared to intact berries were found for white varieties in a previous study (Weißinger et al., 2019a). These findings suggest that removal of epicuticular wax layers does not have the same effect on offspring performance as pre-damage of the grape berry skin. Thus, developmental success and emergence rates of *D. szuzukii* offspring obviously do not exclusively depend on physical properties of epicuticular waxes but might be also affected by the barrier properties of cutin and intracuticular waxes.

Variation in offspring performance among dewaxed “Regent” and dewaxed berries of the other varieties may be explained by slight differences in mechanical and chemical features of the internal pulp texture. Traits such as pulp elasticity and consistency have been related to offspring emergence success, for example, by affecting the gas exchange of *D. suzukii* eggs and larvae (Tonina et al., 2020). In addition, subtle differences in fruit chemical composition, such as sugar content, acidity, nutrient content and specialized (secondary) plant compounds of the inner fruit may have influenced the larval development in different grape berries (Rodriguez-Saona et al., 2019; Little et al., 2020b; Tonina et al., 2020). Sugar content has been found to correlate with *D. suzukii* oviposition and performance (Ioriatti et al., 2015; Lee et al., 2011). However, since only ripe berries with comparable Brix levels were used in our bioassays (Table 1), sugar content does not explain the observed differences.

Micromorphological analyses of grape surfaces revealed that all *V. vinifera* varieties were densely covered with a bloom of epicuticular wax crystals (Figure 4). They had a distinct crystal architecture with numerous irregular platelets (nomenclature following Barthlott et al., 1998), that showed different serrated and fissured fine structures in a random orientation (Figure 4). A similar epicuticular wax ultrastructure and morphology has been described for “Pinot Noir” (Comménil et al., 1997) and other *V. vinifera* varieties (Casado and Heredia, 2001; Rogiers et al., 2004). Plant wax crystals have a protective function against various herbivorous insects, among others due to their anti-adhesive properties (Gorb and Gorb, 2009, 2017). For example, microroughness and easily breaking structures of wax crystals can have a deteriorative effect on the attachment of insect tarsae or eggs and hence on insect locomotion, feeding or oviposition on plant surfaces (Eigenbrode, 2004; Müller, 2008; Gorb and Gorb, 2017). From behavioral recordings we have clear evidence that the surface structure of berries strongly impacts the attachment and locomotion of *D. suzukii* (Breuer and Wyss, 2015; Supplementary Video 1 on berry of a red variety). The females of *D. suzukii* have enormous difficulties to get a proper grip with their tarsae on the intact grape berry surface. Especially during the oviposition process they readily slip off with their tarsae (Breuer and Wyss, 2015; Supplementary Video 1). Consequently, the piercing of the berry skin as well as oviposition may take much longer and a strong force is needed on berries compared to other fruit surfaces. We assume that the removal of epicuticular waxes in “Regent”, “Acolon”, “Pinot Noir”, and “Pinot Blanc” may have resulted in a less slippery surface and better grip for *D. suzukii* tarsae, whereas this does not seem to be the case in “Müller-Thurgau” (Figures 2D,I). Wax crystals of “Müller-Thurgau” are quite fine, rather filamentous and relatively flat, as also recently shown by Arand et al. (2021). Thus, *D. suzukii* may already find a better grip on intact “Müller-Thurgau” berries than on intact berries of the other varieties, whose crystals appear much stronger.

The chemical composition of both epi- and intracuticular waxes was quite different among the grape varieties (Figures 5, 6). Variation in the composition of plant cuticular waxes has been shown before, however, it should be noted that these studies did not or only rarely distinguish between the chemical composition of intra- and epicuticular waxes. So far, research has mostly focused on comparing either the composition of the complete cuticular waxes between varieties within a species (Pensec et al., 2014; Rid et al., 2018; Klavins, 2020) or the composition of epi- and intracuticular waxes within one variety or species (Jetter et al., 2006; Koch and Ensikat, 2008). Within all grape varieties studied here, chemical analyses revealed remarkable differences between epi- and intracuticular wax composition of the berries (Figures 5, 6), which is likewise known for leaves of several plant species (Buschhaus and Jetter, 2011; Jetter and Riederer, 2016). The composition of the berry waxes of “Regent” and “Acolon” differed most clearly from the others (Figure 5). As shown previously (Weißinger et al., 2019a), exactly these two red varieties were more susceptible than “Pinot Noir”, “Müller-Thurgau”, and “Pinot Blanc”. Hence, differences in cuticular wax composition between these varieties may be a reason for varietal preferences of *D. suzukii*, but this needs further exploration. However, we cannot exclude that specialized metabolites may be additionally present in the epicuticular waxes of the berries, as known for other plant species (Müller, 2006; Shroff et al., 2015), which may affect the behavior of this fly.

The absolute amount of epicuticular waxes was significantly lower than that of intracuticular waxes (Table 4). Moreover, epicuticular waxes of all varieties were almost entirely composed of very long chain aliphatic acids, alkanols, alkanals and esters, which is in line with previous studies on grapes (Radler and Horn, 1965; Possingham et al., 1967). The most pronounced differences between the two wax layers were found in the triterpenoids, which occurred predominantly in intracuticular waxes while they were missing in epicuticular waxes of all varieties. Among the triterpenoids, oleanolic acid was found to be the main component in all intracuticular waxes, as previously reported for other *V. vinifera* varieties (Radler, 1965; Pensec et al., 2014). The cutin was mainly composed of hexa- and octadecanoic acid derivatives, with ω-hydroxy-9,10-epoxy-octadecanoic acid being the main compound. The cutin amount was quite comparable among varieties within 2017, which may relate to the similar berry firmness. Cutin is known to interact with triterpenoids and to be important for the mechanical strength of the fruit cutin matrix (Tsubaki et al., 2013).

Interestingly, triterpenoids, known to have insecticidal and fungicidal properties (Eigenbrode, 2004; Szakiel et al., 2012), were solely located underneath the wax crystals and thus could not have affected the flies’ preference behavior in intact berries. However, removal of the epicuticular waxes from tested grapes enabled the flies to have direct contact with the intracuticular waxes. Hence, compounds such as oleanolic acid may act as chemical cues for *D. suzukii*, which in turn may explain the increased oviposition found in dewaxed compared to intact berries. Oleanolic acid in grape waxes appears to be a key factor for oviposition of a European grapevine moth species (Rid et al., 2018) and to affect grape susceptibility to gray mold, *Botrytis cinerea*, with higher amounts of oleanolic acid reducing its germination rate (Comménil et al., 1997). Moreover, Cha et al. (2020) recently reported a reduced attraction, oviposition and performance of *D. suzukii* in fruit infected with *B. cinerea*. Accordingly, oleanolic acid may also have an effect on *D. suzukii* performance, which may be related to the very low overall emergence rate found in the present study. Hiding of stimulating compounds beneath the epicuticular surface may be a very important mechanism to reduce herbivore attack (Reifenrath et al., 2005; Städler and Reifenrath, 2009) and may have been less pronounced in “Müller-Thurgau”, for which intact and dewaxed berries were comparably accepted, due to its surface characteristics.

In summary, our results demonstrate that grape varieties differ in the morphology and chemical composition of epi- and intracuticular waxes. Differences in composition among wax layers may explain the preference by *D. suzukii* for dewaxed grapes, while variety-specific differences in wax composition may be related to the specific preferences for certain varieties by female *D. suzukii*. Our findings may have important implications for winegrowers regarding variety selection and grape health.

## Data Availability Statement

The raw data supporting the conclusions of this article will be made available by the authors, without undue reservation.

## Author Contributions

LW, KA, MB, and CM conceived the research plans. MB and CM supervised the experiments. LW harvested the grape berries, measured the penetration force and performed the dual-choice bioassays and wrote the original draft of the manuscript with contributions of all authors. KA performed the chemical analyses. EB and H-HK performed the cryo-SEM. LW and CM statistically analyzed the data and MB and KA contributed to data interpretation. CM supervised and completed the writing and agrees to serve as the author responsible for contact and ensures communication. MB was in charge of project administration and funding acquisition. All authors contributed to the article and approved the submitted version.

## Conflict of Interest

The authors declare that the research was conducted in the absence of any commercial or financial relationships that could be construed as a potential conflict of interest.
