# Supplementary material for: Physical and Chemical Traits of Grape Varieties Influence Drosophila suzukii Preferences and Performance
Source: Front Plant Sci. 2021 Apr 21;12:664636. doi: 10.3389/fpls.2021.664636 (PMC8098983; doi:10.3389/fpls.2021.664636)
Supplement: Supplementary file 1 [file Data_Sheet_1.PDF]

Supplementary Material

1 Supplementary Data

1.1 Supplementary Figures

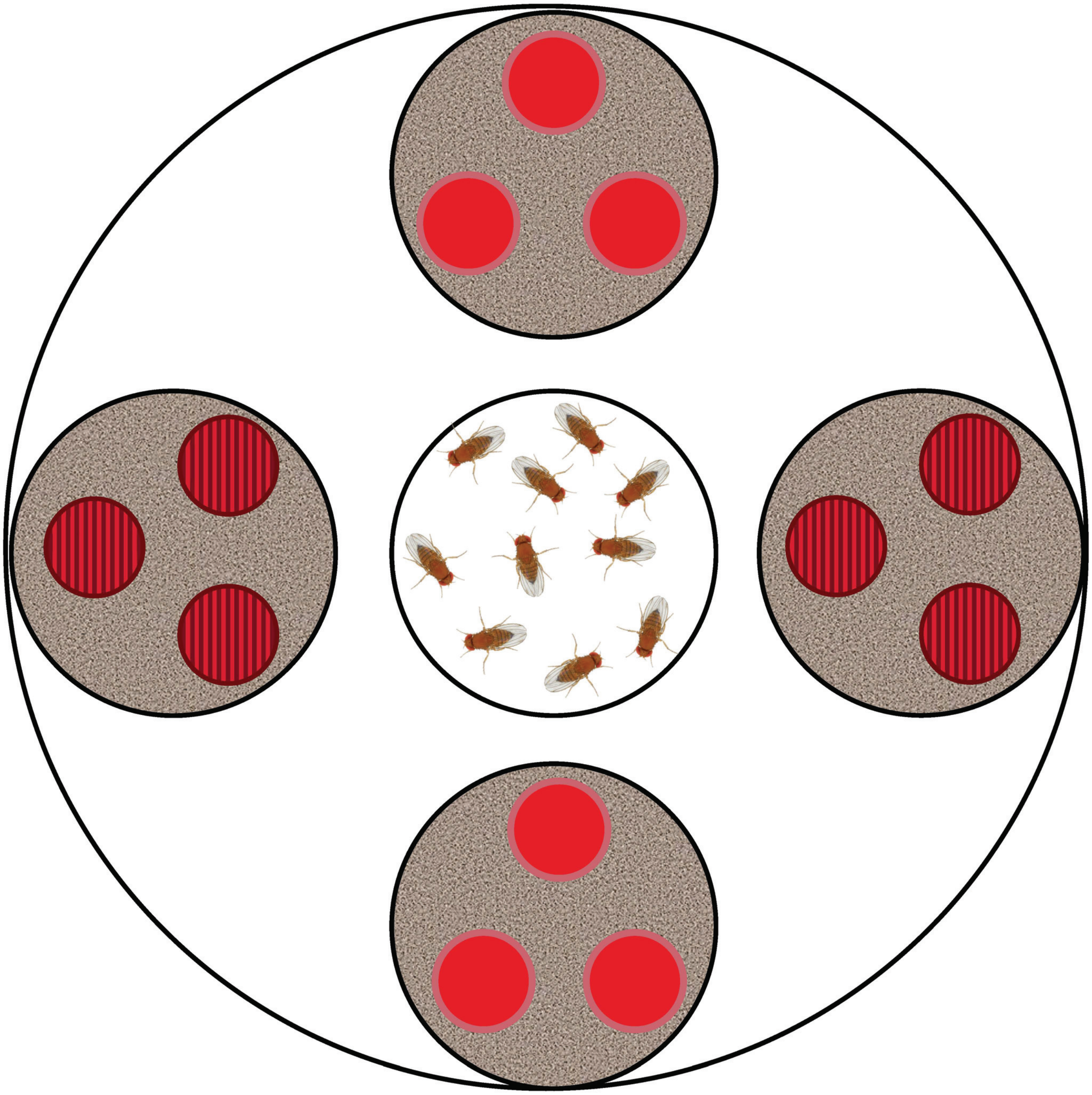

Supplementary Figure 1. Top-view of the test arena used in dual-choice bioassays. Ten female flies were released in the center of each arena. Two times three berries per choice option (berry condition) were placed in two small Petri dishes on opposite sides of the arena. The bases of the test berries

were embedded in sand to prevent the flies from hiding and to keep the berries in place. Modified from Weißinger et al. (2019).

1.2 Supplementary Videos

Supplementary Video 2. Oviposition behavior of female of *Drosophila suzukii*. Sequence of Breuer, M., Wyss, U. (Producers) (2015). Behavior and development of the spotted wing Drosophila *Drosophila suzukii*. Germany: Entofilm. Available from https://www.entofilm.com/
